# Supplementary material for: Drug trends and harm related to new psychoactive substances (NPS) in Sweden from 2010 to 2016: Experiences from the STRIDA project
Source: PLoS One. 2020 Apr 23;15(4):e0232038. doi: 10.1371/journal.pone.0232038 (PMC7179898; doi:10.1371/journal.pone.0232038)

S1 Fig. Box-and-whisker plot showing age distribution of patients in relation to main substance class. In 78% of the cases, a classification into either of the following main substance classes was possible: cannabis or synthetic cannabinoid receptor agonists (Cannabi), ethanol, hallucinogens (Hallucino), benzodiazepines (Benzodi), dissociative drugs (Dissocia), opioids, pregabalin (Pregaba), or stimulants (Stimula). N = number of cases; open point = outside value; filled point = far out value.

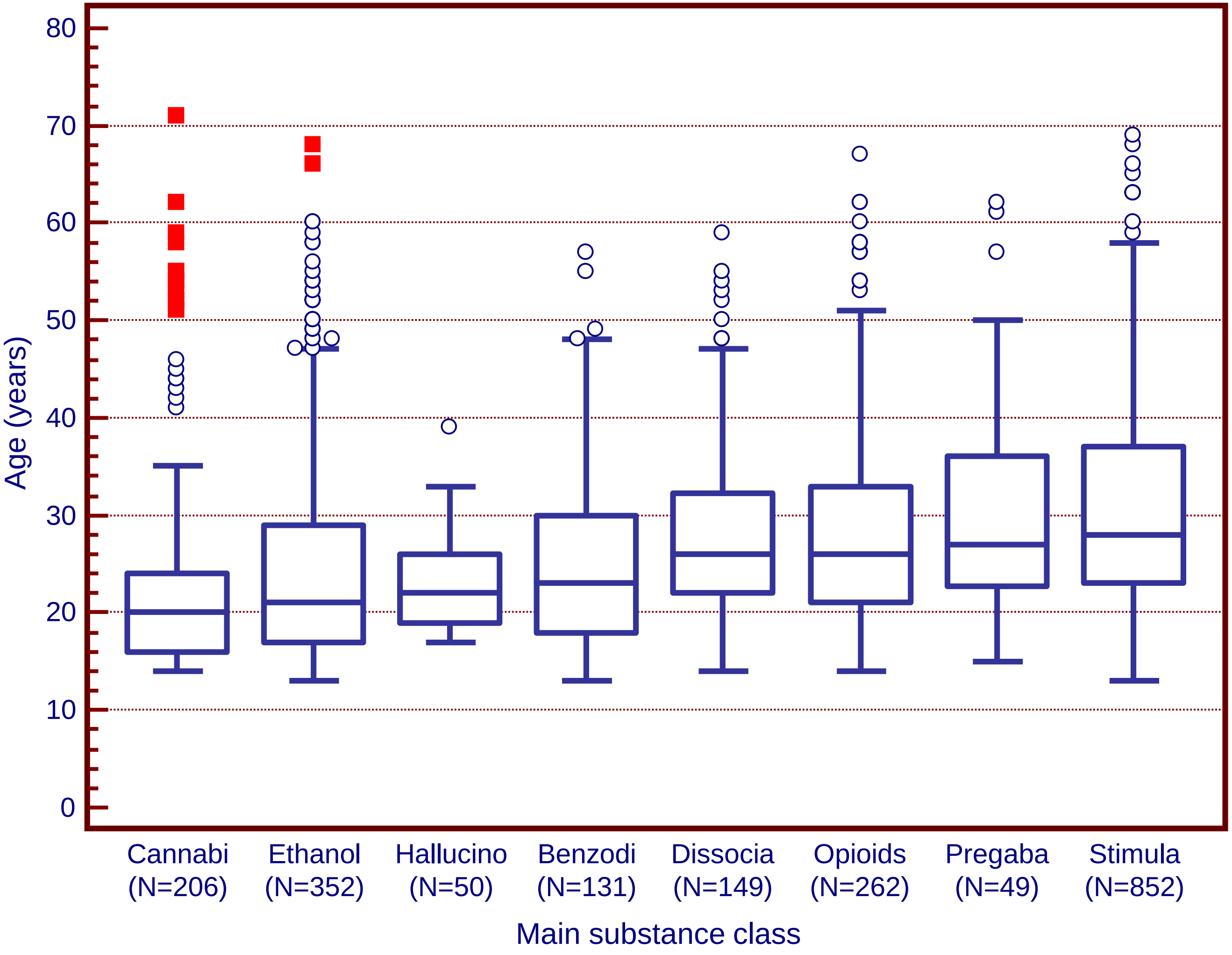

Supplement: S1 Fig — In 78% of the cases, a classification into either of the following main substance classes was possible: cannabis or synthetic cannabinoid receptor agonists (Cannabi), ethanol, hallucinogens (Hallucino), benzodiazepines (Benzodi), dissociative drugs (Dissocia), opioids, pregabalin (Pregaba), or stimulants (Stimula). N = number of cases; open point = outside value; filled point = far out value. (PDF) [file pone.0232038.s001.pdf]
